# Professor Dr. Peter Vaupel zum 80. Geburtstag

**DOI:** 10.1007/s00066-023-02109-2

**Published:** 2023-07-21

**Authors:** Anca-Ligia Grosu, Andreas Thomsen

**Affiliations:** Klinik für Strahlenheilkunde, Department für Radiologische Diagnostik und Therapie, Universitätsklinikum Freiburg, Freiburg, Deutschland

**Figure 1:**
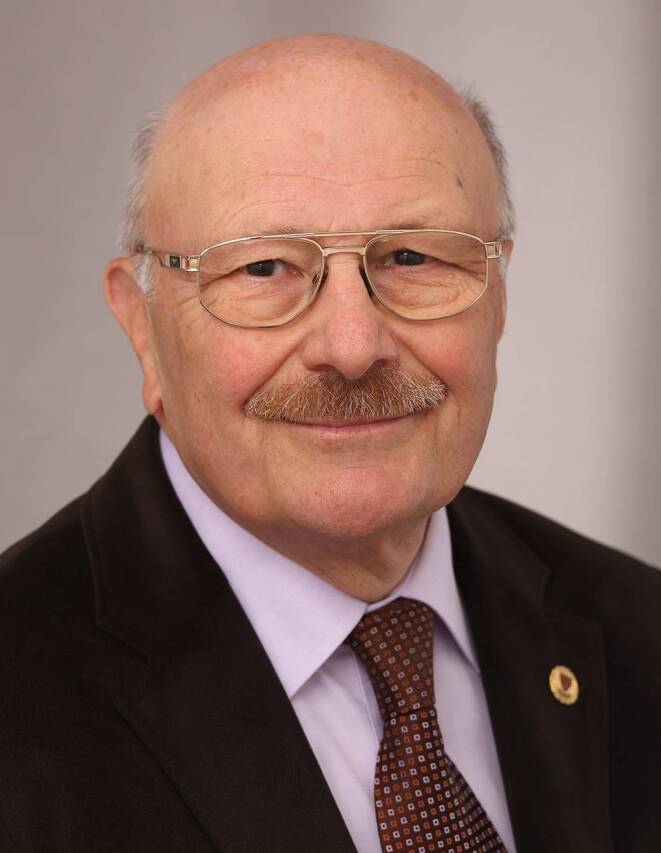

Peter Vaupel (Abb. 1) wurde am 21. August 1943 in Lemberg/Pfalz geboren. Nach dem Besuch des mathematisch-naturwissenschaftlichen Gymnasiums in Pirmasens studierte er von 1963 bis 1968 Medizin an der Johannes Gutenberg-Universität in Mainz. Bereits 1969 wurde er mit einer Dissertationsschrift aus dem Bereich der medizinischen Biophysik promoviert.

Nach der Medizinalassistentenzeit und der Approbation als Arzt nahm er 1970 das Angebot an, als Wissenschaftlicher Assistent an das damalige Physiologische Institut der Mainzer Universität zu wechseln, das von Professor Dr. Dr. G. Thews, einem international hoch angesehenen Atmungsphysiologen, geleitet wurde. Die Forschung des Instituts fokussierte auf die Sauerstoffversorgung lebenswichtiger Organe. Aufgrund dieser inhaltlichen und methodischen Ausrichtung entschied sich Peter Vaupel, die Oxygenierung maligner Tumoren *in vivo *mithilfe zahlreicher, z. T. neu entwickelter Mikrotechniken zu analysieren. Dieses Forschungsgebiet, das für unser Fachgebiet eine kaum zu überschätzende Rolle hat, treibt ihn bis heute an. Seine außergewöhnliche wissenschaftliche Arbeit umfasst mehr als 600 Publikationen, über 700 Vorträge und Kongressmitteilungen sowie die (Mit‑)Herausgeberschaft von 17 Fachbüchern; er gehört zu den international meistzitierten Pathophysiologen. Peter Vaupel habilitierte im Alter von 30 Jahren mit einer Schrift zur Sauerstoffversorgung bösartiger Tumoren. Gleichzeitig wurde er 1974 zum damals jüngsten Professor der Medizinischen Fakultät an der Universität Mainz ernannt. Fünf Jahre später (1979) nahm er die Einladung auf eine Gastprofessur am Department of Therapeutic Radiology, Radiation Biology and Physics Division des Henry Ford Hospital in Detroit (USA) an. Er unterstützte dort nachhaltig den Aufbau einer tumorbiologischen Abteilung. Die Ernennung zum C3-Professor und Leiter der Abteilung für Angewandte Physiologie am Fachbereich Medizin der Universität Mainz erfolgte 1983.

Aufgrund seines hohen wissenschaftlichen Renommees erhielt er 1986 den ehrenvollen Ruf als Full Professor an der Harvard University in Boston. Während seiner Zeit als „Andrew Werk Cook Professor für Strahlenbiologie, Tumorbiologie und Physiologie“ an dieser amerikanischen Eliteuniversität erfolgten Rufe auf C4-Professuren an die Universitäten Münster (abgelehnt) und Mainz. Den Ruf auf den Lehrstuhl für Pathophysiologie im Fachbereich Medizin der Johannes Gutenberg-Universität Mainz nahm er 1989 an.

Die Amtszeit als Ordinarius dauerte bis zur Pensionierung im September 2008. Peter Vaupel setzte sich mit Kraft für eine klinikorientierte Ausbildung der Studierenden im Fach Physiologie ein. Seine Vorlesungen, Seminare und Praktika, die er mit hohem Engagement und pfälzischem Timbre hielt, fanden großen Anklang. Schwierige Sachverhalte konnte er den angehenden Ärztinnen und Ärzten anschaulich und eindrücklich vermitteln. Als Universitätslehrer und Facharzt für Physiologie widmete sich Peter Vaupel auch der wissenschaftlichen Qualifikation von Studierenden und jüngeren Kollegen und Kolleginnen. So betreute er 41 Medizin-Doktoranden, 10 Biologie-Diplomanden und -Doktoranden sowie 11 Habilitanden, 5 davon im Fach Physiologie, 6 weitere in Chirurgie, Gynäkologie, pädiatrischer Onkologie und Strahlentherapie. Auch hieran zeigt sich, dass der wissenschaftliche Input von Peter Vaupel weit über die engeren Fachgrenzen reichte bis hinein in verschiedene Disziplinen der klinischen Medizin. Sein Einsatz für die Mainzer Fakultät ist durch seine langjährige Mitgliedschaft im Fachbereichsrat Medizin sowie seine 10-jährige Tätigkeit als Vorsitzender des Bereichsausschusses Vorklinik wie auch durch Dienste als Prodekan des Fachbereichs Medizin (1997–1999) belegt. Die vorklinische Lehre war Peter Vaupel eine Herzensangelegenheit, bei der er die Verknüpfung der Physiologie mit den benachbarten Disziplinen und der Klinik auf bemerkenswerte Weise meisterte. Dies zeigt sich auch an seiner Mitherausgeberschaft von zwei Lehrbüchern in den Bereichen „Vegetative Physiologie“ (zusammen mit G. Thews) sowie „Anatomie, Physiologie und Pathophysiologie des Menschen“ (zusammen mit G. Thews und E. Mutschler). Beide Bücher gelten als Standardwerke und sind von hoher didaktischer Qualität.

Peter Vaupel engagierte sich in vielfältigen weiteren wissenschaftlichen und berufspolitischen Aufgaben. Er war und ist langjähriges Mitglied im Editorial Board einer Reihe von renommierten Fachjournalen. Zu diesen gehören u. a. *Cancer Research* (über 20 Jahre), *International Journal of Radiation Oncology, Biology, Physics* (16 Jahre), und bereits vor 30 Jahren wurde er auch Mitglied des wissenschaftlichen Beirats unserer Zeitschrift *Strahlentherapie und Onkologie*, in welcher er bis heute als wissenschaftlicher Berater dient. Weiterhin war er von 1990 bis 2003 Mitglied der medizinischen Sachverständigenkommission des Instituts für Medizinische und Pharmazeutische Prüfungsfragen (IMPP, Fachgebiet Pathophysiologie).

Die breit angelegten Aktivitäten von Peter Vaupel wurden durch zahlreiche Ehrungen ausgezeichnet: Boehringer-Ingelheim-Forschungspreis (1974), Vorsitzender der Deutschen Gesellschaft für Mikrozirkulation (1983, 1991), Honorary Master’s Degree (M. A.) der Harvard University (1988), Lund Science Award (1989), Präsident der International Society on Oxygen Transport to Tissue, (ISOTT, 1991/92), Stiftungsrat der Dr. med. h.c. Erwin Braun Stiftung, Basel (seit 1993), Vorsitzender des Stiftungsrats der Dr. med. Wulf Vater Stiftung, Mainz (1996–2021), ordentliches Mitglied der Akademie der Wissenschaften und der Literatur zu Mainz (seit 1998), Vorsitzender der Kommission für Humanforschung an der Akademie der Wissenschaften und der Literatur zu Mainz (1999–2003), Berufung als Mitglied der Kommission für Theoretisch-Medizinische Forschung an der Akademie der Wissenschaften und der Literatur zu Mainz (seit 2003), ESHO-Award (2004), 1st Robert F. Kallman Memorial Lecture der Stanford University Medical School (2004), Lifetime Achievement Awards der IAHOM, Indien (2012, 2020), A. Kovach Memorial Lecture, ISOTT (2012) sowie 2019 die Verleihung der Ehrenprofessur an der Odessa National Medical University. Weiterhin wurden ihm von der Scientific Association of Swiss Radiation Oncology (SASRO, 2004) und der Deutschen Gesellschaft für Radioonkologie (DEGRO, 2006) Ehrenmitgliedschaften verliehen [1].

Peter Vaupel steht in direktem Austausch mit Klinikern und in Kooperation mit Biologinnen und Biologen, um das große und wichtige Thema „Tumorpathophysiologie, Tumorhypoxie und Tumormikromilieu“ intensiv weiter zu bearbeiten – nicht nur, um neue wissenschaftliche Erkenntnisse zu generieren, vielmehr auch, um klinisch nutzbare Beiträge zu liefern, die in Zukunft dem Krebskranken von Vorteil sind. Es ist bekannt, dass Peter Vaupel in jüngeren Jahren mit großer Freude praktische Medizin betrieben hat. Vielleicht ist auch hier eine Triebfeder für seine Forschung zu sehen, die ihn in der klinischen Onkologie und Strahlentherapie zu einem weltweit hoch anerkannten Wissenschaftler und Gelehrten hat werden lassen.

2008–2019 war er als Gastprofessor für Tumorpathophysiologie und Strategieberater an der Klinik für Radioonkologie und Strahlentherapie sowie als Expert Lecturer im Masterstudiengang „Radiation Biology“ am Klinikum rechts der Isar der TU München tätig.

Seit Oktober 2019 sind wir in der glücklichen Situation, dass Peter Vaupel als Gastprofessor an unserer Klinik für Strahlenheilkunde am Universitätsklinikum Freiburg mitarbeitet.

Ganz besonders sind ihm die folgenden Themen eine Herzensangelegenheit, die er bis heute unermüdlich bearbeitet und voranbringt:

- Die Tumorpathophysiologie und das Tumormikromilieu mit Schwerpunkt auf der Tumorhypoxie
- Die biophysikalischen, zellulären und molekularen Mechanismen, welche die maligne Progression von hypoxischen Tumoren sowie deren immunologische und therapeutische Resistenz bewirken
- Die Suche nach Möglichkeiten zur Überwindung der Tumorhypoxie, insbesondere die milde lokale Hyperthermie als „Partnermodalität“ für ionisierende Strahlen. Die erfolgreiche Etablierung und klinische Anwendung der Oberflächenhyperthermie in unserer Klinik begleitet Peter Vaupel seit 2018.

Als Freund, Mensch und Mitstreiter schätzen wir Peter Vaupels Scharfsinnigkeit und die Begabung, regelmäßig richtige und wichtige Fragen zu stellen, verbunden mit seiner pfälzischen Bodenständigkeit, seinem Humor und einem besonderen Einfühlungsvermögen. Wir wissen von seiner Geselligkeit, seiner Lust zum Wandern in freier Natur. Diese Leidenschaft teilt er mit seiner lieben Frau Lieselotte, die seit 2012 als Erste Vorsitzende der Mainzer Hospiz-Gesellschaft ehrenamtlich tätig ist. Für die Deutsche Gesellschaft für Radioonkologie gratulieren wir Peter Vaupel zu seinem 80. Geburtstag von ganzem Herzen.

Wir freuen uns auf weitere spannende Gespräche und gemütliche abendliche Runden mit ihm und seiner lieben Frau am Rande wissenschaftlicher Kongresse. Wir wünschen ihm den Erhalt seiner unermüdlichen Schaffenskraft, daneben aber auch Zeit für die geliebten Reisen und Wanderungen, weiterhin lebendige Kontakte mit weltweit verteilten Freunden, viel Freude an den Früchten seines Erfolgs und gesundheitlich alles Gute.

*Anca-Ligia Grosu und Andreas* *Thomsen*

Klinik für Strahlenheilkunde, Universitätsklinikum Freiburg
